# Does the serum E2 level change following coasting treatment strategy to prevent ovarian hyperstimulation syndrome impact cycle outcomes during controlled ovarian hyperstimulation and in vitro fertilization procedure?

**DOI:** 10.4274/tjod.48751

**Published:** 2014-09-15

**Authors:** Ömer Hamid Yumuşak, Serkan Kahyaoğlu, Ayşe Seval Özgü Erdinç, Saynur Yılmaz, Yaprak Engin Üstün, Nafiye Yılmaz

**Affiliations:** 1 Zekai Tahir Burak Women’s Health Education and Research Hospital, Clinic of Reproductive Endocrinology, Ankara, Turkey; 2 Zübeyde Hanım Women’s Health Education and Research Hospital, Clinic of Obstetrics and Gynecology, Ankara, Turkey; 3 Bozok University Faculty of Medicine, Department of Obstetrics and Gynecology, Yozgat, Turkey

**Keywords:** Ovarian hyperstimulation syndrome, coasting, cycle outcome, in vitro fertilization

## Abstract

**Objective:**

Ovarian hyperstimulation syndrome (OHSS) remains as a clinical problem for hyperresponder patients during controlled ovarian hyperstimulation and in vitro fertilization (COH-IVF) procedure. Herein, we aimed to evaluate the COH-IVF outcomes in hyperresponder patients managed with coasting treatment strategy for OHSS prevention regarding the establishment of clinical pregnancy as an endpoint of the treatment cycle.

**Materials and Methods:**

The medical records related to the COH-IVF outcome in 119 hyperresponder patients who have exhibited a serum estradiol level greater than or equal to 3000 pg/mL were evaluated. The study was conducted on a total of 119 patients, 98 of whom have been treated by coasting or coasting with GnRH antagonist co-treatment strategies, while the remaining 21 women (control group) have not been managed with coasting treatment. The COH and IVF-ET outcomes in the 119 patients were compared based on the coasting treatment situation.

**Results:**

Among the women who received coasting treatment, the number of patients demonstrating E2 level decrement and also E2 level decrement rate after coasting were similar between patients with and without clinical pregnancy. Total gonadotropin dose, 2PN number, embryo number, and fertilization rate were significantly higher in the patients with a clinical pregnancy.

**Conclusion:**

The coasting treatment is a clinically useful preventive strategy for OHSS avoidance. GnRH antagonist co-treatment decreases the duration of coasting although any detrimental or ameliorating impact of this effect on pregnancy rates have not been seen. The E2 level decrement or increment following coasting treatment seems not to be related to cycle outcomes.

## INTRODUCTION

Ovarian hyperstimulation is characterised by cystic enlargement of the ovaries, gastrointestinal symptoms, hypovolemia, hemoconcentration, thromboembolic events, respiratory distress and renal failure^(1,2)^. The incidence of moderate form is approximately 3%-6%, where as the potentially life-threatening severe forms occur in 0.1%-3% of all cycles^(2)^. The main risk factors of the syndrome are young age, polycystic ovary syndrome (PCOS), high absolute or rapidly raising serum estradiol levels and high basal antimullerian hormone (AMH)^(3,4)^. The pathophysiology of Ovarian hyperstimulation syndrome (OHSS) depends on increased capillary permeability with fluid shift from intravascular compartment to the extravascular area^(2,5,6)^.

OHSS remains as a clinical problem for hyperresponder patients during controlled ovarian hyperstimulation for in vitro fertilization (COH-IVF). The endogenous or exogenous hCG exposure in patients with a large number of follicles (≥20) on both ovaries and E2 concentration >3000 pg/ml is the initiating factor for OHSS. Several treatment strategies with different clinical outcomes have been recommended for OHSS prevention^(7,8)^.

Coasting has been widely used successfully in IVF centers since the 1980s as an effective method to avoid OHSS^(9,10,11,12)^. Coasting is withholding gonadotropin stimulation and delaying the hCG trigger until serum estradiol levels drop to a safe level while GnRH agonist administration continues. It may lower the incidence and severity of OHSS in high risk patients but does not totaly eliminates the risk of OHSS^(13)^. Despite many studies done in the literature, there is no consensus for a specific coasting protocol on how to apply it, such as when to start coasting, duration of coasting or a threshold for the pecentage of serum estradiol level decrement that would not compromise the IVF outcome.

In this study, we evaluated the COH-IVF outcomes of the hyperresponder patients managed with coasting for OHSS prevention regarding the establishment of clinical pregnancy as an endpoint of the treatment cycle.

## MATERIALS AND METHODS

This retrospective cohort study was undertaken in the assisted reproduction unit of a tertiary education and research hospital. All investigations related to this study have been approved by the local ethical committee and that consent has been obtained from all patients. The database of all patients who underwent ovarian stimulation for assisted reproduction between 2008 and 2013 was retrospectively examined. The medical records of 119 hyperresponder patients who have exhibited a serum estradiol level greater and equal to 3000 pg/mL have been evaluated following COH treatment for an ART procedure. The study was conducted on 119 patients totally, 98 of whom have been treated by coasting. Twenty one patients who have not been managed with coasting treatment have been selected as the control group. COH procedures of the patients were commenced with antagonist protocol for all patients.

During antagonist protocol, patients have received recombinant FSH starting on days 2 or 3 and 0.25 mg cetrorelix (Cetrotide; Asta Medica, Frankfurt, Germany) was administered daily when two or more follicles reached 14 mm in diameter. Human menopausal gonadotropin (hMG) was adminstered to individual patients when clinically indicated based on the ovarian response to COH treatment. The doses of hMG and recombinant FSH have been adjusted according to the ovarian response for both groups until the day of final oocyte maturation by using hCG. One or two days of coasting strategy was commenced for the study group preceding the ovulation trigger regardless of the serum estradiol levels on hCG day. In our practice, when estradiol levels were greater than 3000 pg/mL in the presence of at least 20 follicles, each measuring ≥10 mm in diameter with ≥20% of them of diameter ≥15 mm, recombinant FSH administration was discontinued. For the control group and the study group (following the coasting application), recombinant hCG (250 micrograms sc., Ovitrelle, Serono, İstanbul, Turkey) was administered when at least two leading follicles reached a mean diameter of 17 milimeters. Thirty six hours after hCG injection transvaginal oocyte retrieval was performed. Following oocyte retrieval, metaphase 2 oocytes were reviewed and day 3 embryo transfer (ET) was performed via using pelvic ultrasonography for all patients. Luteal phase support was applicated by vaginal progesterone (Crinone 8% gel, Serono, İstanbul, Turkey) supplementation twice a day until menstruation or for 12 weeks following ET procedure in case of a clinical pregnancy establishment. The presence of a gestational sac with accompanying fetal heartbeat by ultrasound at least 4 weeks after ET was defined as a clinical pregnancy. The COH and IVF-ET outcomes of 119 patients were compared based on the coasting receivement status.

Statistical analysis was performed by using IBM SPSS Statistics Software (19.0, SPSS Inc., Chicago, IL, USA). The categorical variables were compared with Fisher’s exact or Pearson chi-square tests when available. Kolmogorov-Smirnov test was used to determine the normality of the distributions of data. The continuous variables were presented as mean± standard deviation values and compared by using the independent samples t test when distributed normal. Mann-Whitney U test was used when the results were not found to be distributed normal or for comparison of non-parametric data. The potential negative influence of coasting was evaluated in a multivariate logistic regression models considering biochemical pregnancy rate as a dependent variable, after testing each factor in a univariate analysis. Odds ratio (OR) and 95% confidence intervals (CI) were estimated, adjusting the multivariate analysis for confounding variables (using chi-squared test of heterogeneity to control for confounders) and excluding variables with high collinearity. P values <.05 were considered statistically significant.

## RESULTS

The COH-IVF cycle otcomes of the study population have been demonstrated in (Table 1). The mean E2 level on hCG day, gonadotropin stimulation days, p level on hCG day, fertilization rate for the coasting group were significantly lower than control group. All other COH-IVF cycle outcome parameters including retrieved oocye numbers and clinical pegnancy rates were similar between the study and control groups. The comparison for IVF-ICSI outcomes of the coasting group patients regarding the pregnancy occurence as endpoint has been presented in (Table 2). The duration of the coasting treatment was significantly lower in the patients who achieved a clinical pregnancy. Among patients who received coasting treatment, the number of patients demonstrating E2 level decrement and also E2 level decrement rate after coasting was similar between patients with and without clinical pregnancy. Total gonadotropin dose, 2PN number, embryo number and fertilization rate were significantly higher in the patients with a clinical pregnancy. All other COH-IVF cycle outcome parameters were similar between the patients with and without a clinical pregnancy. The rate of coasting with a GnRH antagonist cotreatment was also similar between these groups. Positive pregnancy test, the outcome variable studied in a logistic regression model, was correlated to coasting treatment status, age, basal FSH, number of oocytes retrieved, number of embryos transferred and blastocyst-stage embryo development (coasting treatment status: RR=7.6, 95% CI:1.73-33.49, p=0.007; age: RR=0.93, 95% CI: 0.85-1.02, p=0.14; basal FSH: RR=0.94, 95% CI:0.71-1.23, p=0.66; number of oocytes retrieved: RR=1.03, 95% CI:0.94-1.12, p=0.47; number of embryos transferred: RR=0.73, 95% CI:0.46-1.15, p=0.18; blastocyst-stage embryo development: RR=0.22, 95% CI:0.07-0.67, p=0.007) (Hosmer and Lemeshow Test p value was 0.49 that demonstrated the logistic regression model’s validity).

## DISCUSSION

In the coasting group, the mean E2 level on hCG day, gonadotropin stimulation days, the number of gonadotropin ampules used and fertilization rate were significantly lower than control group. However metaphase 2 oocytes, total oocytes retrieved, 2PN number, embryo grades, number of embryos transferred, clinic pregnancy and OHSS rates were similar between coasting cycles and the control group. In this study, cotreatment with GnRH antagonist accompanying with coasting has also not been found to be related to clinical pregnancy probability. The coasting duration (days) of patients who achieved clinical pregnancy was significantly lower than the patients who have not achieved a clinical pregnancy. This result supports the usual consideration of the fact that higher coasting duration decreases the pregnancy rates as mentioned in the previous studies.

The duration of coasting that is effective in reducing the incidence of OHSS without compromising the cycle outcomes has not been established yet. Most studies showed that withholding gonadotropins up to 3 days did not affect the cycle outcomes^(9,13)^. However other studies demonstrated that prolonged coasting for >3 days compromise the IVF outcomes. Ulug et al. found that coasting for more than 3 days reduced the implantation and pregnancy rates while oocyte and embryo quality did not appear to be affected^(14)^. In a large cohort of patients, Mansour et al. reported that coasting more than 3 days reduced significantly the mean number of oocytes retrieved, the implantation and clinical pregnancy rates, but on the other hand the incidence of OHSS was reduced to 0.13% in all stimulated cycles and to 1.3% in patients at risk for OHSS^(15)^. Owj et al. concluded their study as prolonged coasting (>3 days) had a negative effect on the number and quality of oocytes^(16)^. Waldenstrom et al. showed that coasting more than 3 days decreases the number of oocytes retrieved and the pregnancy rates. They claimed that withholding gonadotropins reduces the LH receptor levels and if the duration of this period lasts longer, the LH receptor levels decreases severely. The follicles with severely decreased LH receptors respond poorly to exogenous hCG so the oocytes with mature sized follicules will not complete the final maturation. The immature oocytes will stick to the follicule wall and this will cause reduction in the number of oocytes retrieved^(17)^. Nardo et al. compared the coasting cycles with 1-3 days with more than 3 days. Coasting more than 3 days reduced the number of oocytes retrieved and decreased the implantation rate. There was no difference in pregnancy and live birth rate. The lower implantation rate was assosiated with negative effect on endometrial receptivity^(18)^. Isaza et al. suggested that if the duration of coasting was more than 5 days or if a severe fall in the level of estradiol (<1000 pg/ml) observed, the oocyte quality might be affected^(19)^. In our study we found that the duration of coasting was significantly lower in the patients who achieved a clinical pregnancy just like the previous studies. Besides, based on the logistic regression analysis results, we demonstrated that coasting strategy increased the clinical pregnancy achievement probability more than the expectant (control group) management strategy. Interestingly, lower clinical pregnancy rates have been found for blastocyst transfer in our study that might be a result of longer coasting duration among patients who received blastocyst transfer. When compared with the control group; the total gonadotropin dose, 2PN number, embryo number and fertilization rates were significantly higher among the patients who received coasting treatment with a clinical pregnancy that demonstrates the clinical effectiveness of coasting strategy.

Gonadotropins upregulate the gonadotropin receptors and inhibit the granulosa cell apoptosis of small immature follicules. The FSH concentration decrease due to withholding gonadotropins that induce apoptosis of small immature follicules, which are more sensitive to FSH paucity, results with reduced levels of the vasoactive substances responsible for capillary permeability and fluid extravasation^(20)^.

There is no consensus for a specific coasting protocol on how to apply it, despite many studies done in the literature. In most studies the estradiol cutoff value for withholding gonadotropins is between 2500 and 4000 pg/ml^(9)^. We started coasting in our IVF cycles when the serum estradiol levels were >3000 pg/ml. Mansour et al. started coasting according the size of follicules instead of the level of estradiol. When the leading follicules reached 16 mm in size, they withhold gonadotropins and waited estradiol levels to fall under 3000 pg/ml for hCG administration^(15)^. Al-Shawaf et al. suggested that falling in FSH levels by 25% daily during coasting period and a decline in serum FSH to 5 mIU /ml was safe for hCG injection^(10)^.

It is still a controversial issue whether the rate of decrease in serum estradiol level compromises IVF outcome. Many authors similarly found that the rate of estradiol level decrement during coasting treatment did not effect the pregnancy and implantation rate^(13,14)^. However in some studies cycle cancellation was recommended when the rate of estradiol level decreased by more than >20% after hCG injection^(21)^. Abdalla et al. demonstrated that neither E2 increase nor E2 decrease following coasting treatment has a negative effect on implantation, miscarriage, or live birth rates, except extremely low (<1.000 pmol/L) or high (>20.000 pmol/L) serum E2 levels at hCG triggering^(22)^. In our study we also have not found a relationship between E2 level decrement or increment following coasting treatment and clinical pregnancy occurance.

GnRH antagonist salvage has been used for patients with high serum estradiol levels at risk of developing OHSS. Gustofson et al. observed that with GnRH antagonist cotreatment the estradiol levels sharply decreased to a safe level without affecting negatively on oocyte maturation, embryo quality and fertilization rates^(23)^. In a prospective randomized study Aboulghar et al. compared the effect of GnRH antagonist cotreatment with coasting among the patients with long GnRH agonist protocol. In the antagonist arm the mean number of oocytes retrieved and high quality embryos was significantly higher than in the coasting group. There were more days of coasting as compared with days of antagonist administration. There were no significant differences in the clinical pregnancy and multiple pregnancy rates between the two groups^(24)^. We found that the (+) and (-) pregnancy rates in the patients who received antagonist cotreatment were comparable (26.2% vs. 39.3%). Also fertilization rate, 2PN and embryo number was higher in the patients who are pregnant.

In conclusion, the coasting treatment is a clinically useful preventive strategy for OHSS avoidance. GnRH antagonist cotreatment decreases the duration of coasting although any detrimental or ameliorating impact of this effect on pregnancy rates has not been seen. The E2 level decrement or increment following coasting treatment seems not to be related to cycle outcomes.

### Declaration of Interest

The authors report no declarations of interest. There remain no relevant potential conflicts of interest related to this original article. Also, there remains no affiliation with any organization with a financial interest, direct or indirect, in the subject matter or materials discussed in the manuscript (such as consultancies, employment, paid expert testimony, honoraria, speakers bureaus, retainers, stock options or ownership, patents or patent applications or travel grants. We did not receive any funding and/ or financial support from any commercial or other association for this study. Medical writing of this manuscript has been completed by the authors own.

## Figures and Tables

**Table 1:**
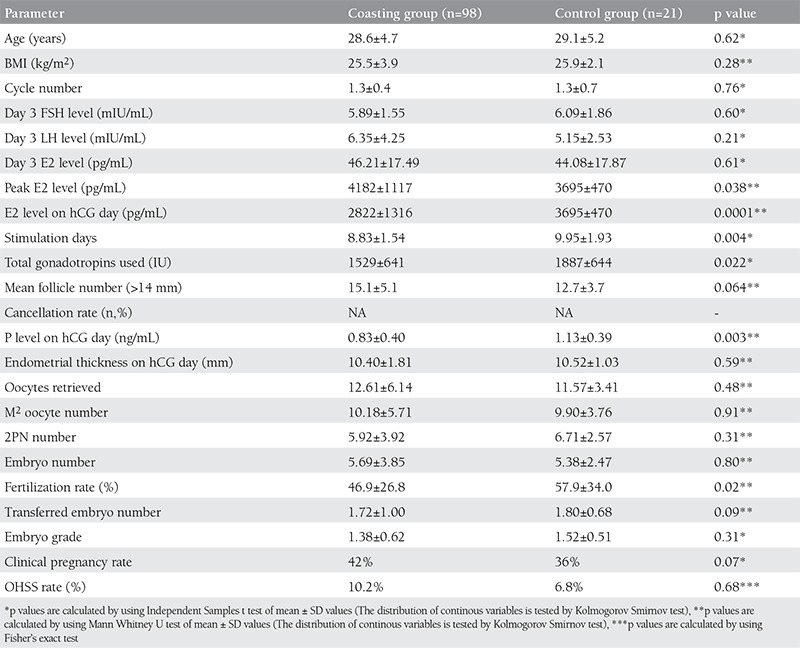
The comparison for IVF-ICSI outcome of the coasting group and the control group (n=119)

**Table 2:**
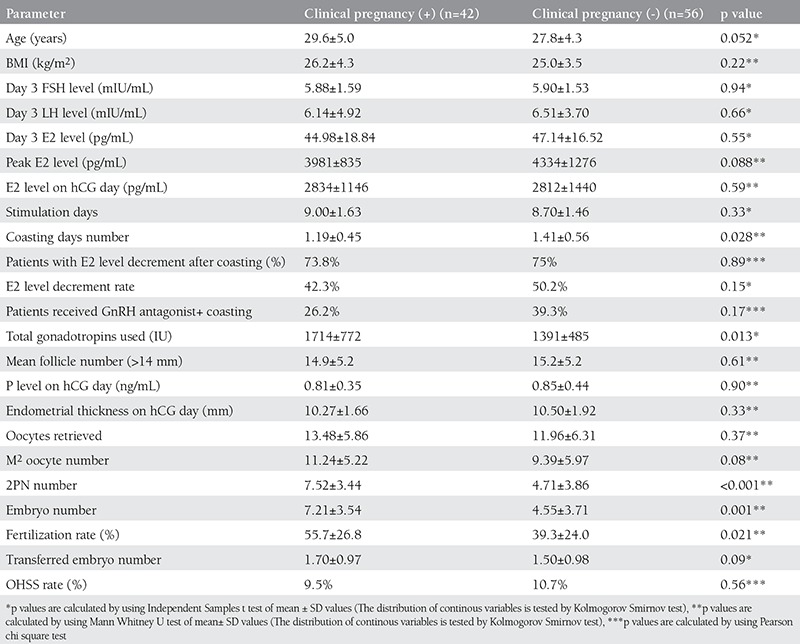
The comparison for IVF-ICSI outcomes of the coasting group patients regarding the pregnancy occurence as end-point (n=98)
